# Development of a Duplex Digital PCR and Validation on eDNA Water Samples for Monitoring of the Asian Swamp Eel (*Monopterus albus*/*Javanensis*) and Bullseye Snakehead (*Channa aurolineata*/*Marulius*) in Florida, USA, Freshwater Ecosystems

**DOI:** 10.1002/ece3.73088

**Published:** 2026-02-17

**Authors:** Melody Bloch, Eric Suarez, Melissa A. Miller, Sergio A. Balaguera‐Reina, Cynthia A. Fussell Persaud, Kevin A. Olejniczak, Ericka E. Helmick, Frank J. Mazzotti, Brian W. Bahder

**Affiliations:** ^1^ Fort Lauderdale Research and Education Center, Department of Entomology and Nematology Institute of Food and Agricultural Sciences University of Florida Davie Florida USA; ^2^ Fort Lauderdale Research and Education Center, Department of Wildlife Ecology and Conservation, Institute of Food and Agricultural Sciences University of Florida Davie Florida USA

**Keywords:** conservation, environment, fish, invasive species, management

## Abstract

Invasive species are a significant threat to a variety of ecosystems in Florida, with freshwater habitats being of particular concern. Two nonnative species of fish with established populations that are priority organisms for monitoring and management programs are the bullseye snakehead (BS; *Channa* species complex) and Asian swamp eel (ASE; *Monopterus* species complex). Recent technological advances have seen the emergence of environmental DNA (eDNA) analysis as a useful tool for the detection and monitoring of target organisms and to assess removal efforts. In this study, a duplex digital polymerase chain reaction (dPCR) was developed, optimized, and validated on control samples and field samples from locations with documented occurrences of target organisms. The assay developed, which allows simultaneous detection of both fish species when present in a sample, demonstrated highly efficient amplification for the corresponding target species with individual assays failing to cross‐amplify. Under controlled conditions, high levels of eDNA were detected as early as five minutes post‐introduction of BS to water. Additionally, field eDNA samples yielded varying levels of positive amplification for both species based on comparison of fluorescence levels to positive controls (both tissue extract and plasmids with appropriate inserts). These data indicate that through careful assay design and stringent parameter optimization, eDNA results obtained for monitoring of these two fish species can be a viable and cost‐effective strategy to detect the presence of these species simultaneously as well as to evaluate the success of removal efforts.

## Graphical abstract

The Asian swamp eel and bullseye snakehead are two invasive organisms in Florida of ecological concern. In this study, a multiplex digital polymerase chain reaction (dPCR) assay is developed, optimized, and validated on water samples for environmental DNA (eDNA) analysis and monitoring.

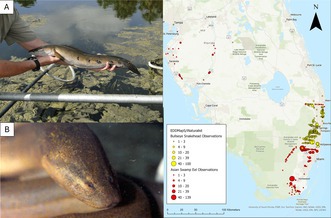

## 1 Introduction

Florida, USA, is a region with high levels of biological invasions and is prone to establishment of introduced species primarily due to its mild temperate to subtropical climate. While organisms representing all taxa present unique challenges to different systems, freshwater fish are of particular concern due to their potential to disrupt aquatic ecosystems, including the Everglades, which is comprised of freshwater and estuarine habitats. Multiple federal and state conservation areas have been established to safeguard the ecological functions and biodiversity of this unique landscape.

**FIGURE 1:**
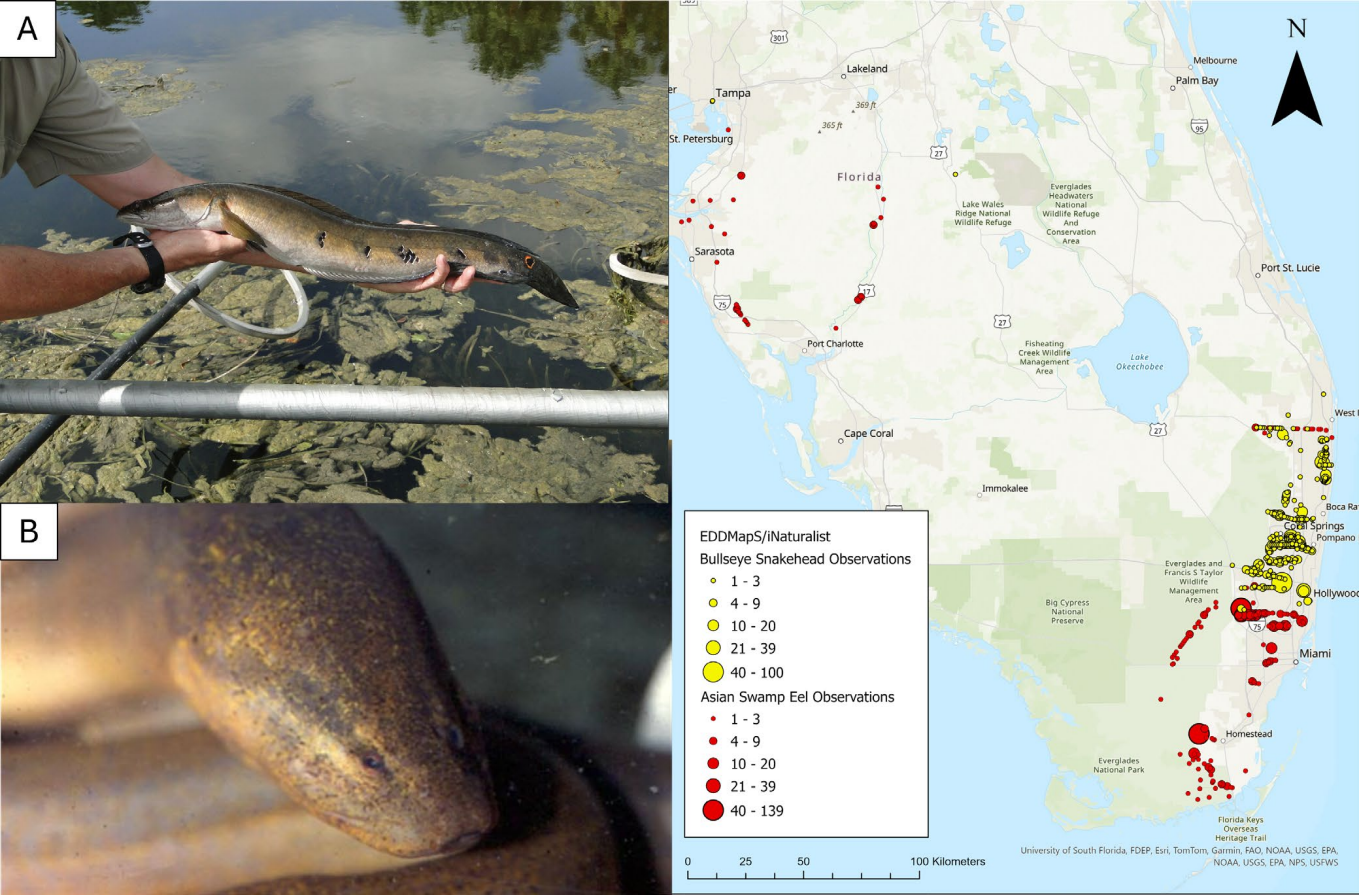
Specimens of the bullseye snakehead (BS) (A) and Asian swamp eel (ASE) (B) collected from Florida, USA, and their current distribution in the state; yellow = BS and red = ASE.

Over time, almost 200 species of introduced freshwater fish have been reported from Florida, with over 45 species currently established (reproducing) in natural (e.g., marshes) or human‐made (e.g., canals) areas (Robins et al. 2019). Two fish species of significant concern are the bullseye snakehead ( *Channa marulius* complex; BS) and the Asian swamp eel ( *Monopterus albus* complex; ASE) (Figure 1). The bullseye snakehead was first documented in 2000 (Shafland et al. 2008) and the Asian swamp eel was first documented in Florida in 1997 (Collins et al. 2002), with three distinct clades/haplotypes documented in the state. Both species are labeled as complexes because of the taxonomic confusion surrounding the variety of specimens seen throughout Florida. The *Channa* complex comprises *C. aurolineata* based on recently established species‐level differences (Nico et al. 2022) and *C. marulius*. The validity of these two species being distinct is difficult to discern due to well below average COI genetic differences observed (Nico et al. 2022) and ambiguity surrounding this distinction is why they will collectively be treated as BS throughout the manuscript. Additionally, the *Monopterus* complex comprises *M. albus* and *M. javanensis* , and it is not clear the true status of each of these (Collins et al. 2002), but will be collectively referred to as ASE throughout the article.

Bullseye snakeheads are relatively large, carnivorous, air‐breathing fish native to southern Asia (Benson et al. 2018). Like other snakehead species (*Channa* spp.), the bullseye snakehead can survive out of water and in hypoxic waters, allowing it to persist during low water or drought conditions. It can potentially move short distances overland to more suitable habitats (Graham 1997; Bressman 2022; Nico et al. 2022). Asian swamp eels are protogynous hermaphroditic, air‐breathing, carnivorous, eel‐like fish (Graham 1997) native to eastern and southern Asia (Hill and Watson 2007). This species is capable of short overland (where shallow water is present) movements (Liem 1987), which may enhance its ability to inhabit new bodies of water where conditions are more favorable (Shafland et al. 2010).

Analysis of environmental DNA (eDNA) can aid in the detection of invasive wildlife, improve early detection, inform occupancy modeling, help determine invasion fronts, and provide managers a method to monitor and assess eradication efforts (Hunter et al. 2015, 2019; Orzechowski et al. 2019; Morisette et al. 2021; Keller et al. 2022; Carim et al. 2019). Different approaches can be utilized depending on the desired management outcome and the questions asked. For example, metabarcoding is a useful approach if the local species assemblage is unknown and the objective is to determine which species of fishes (or other taxonomic groups of interest) are present in a defined location (Darling Deiner et al. 2017; Tsuji et al. 2022). However, if the goal of a program is to monitor for a specific species (i.e., target species) or multiple species that are known to cohabit an area (or that have theoretically been eradicated, requiring validation) then the utility of species‐specific assays coupled with digital polymerase chain reaction (dPCR; Miller et al. 2024) is more practical due to the significantly higher level of sensitivity obtained via dPCR. The utility of eDNA analysis for species of fish has been demonstrated for evaluating general biomass (Rourke et al. 2022), relative abundance (Lacoursière‐Roussel et al. 2016; Doi et al. 2017), and species composition (Sakata et al. 2020).

Despite the power of these technologies, eDNA has yet to be widely deployed as a detection tool for invasive species management. This is in part due to lack of confidence in eDNA detections due to the potential for false positives (failure to discern between a true reading and procedure noise) and false negatives (fail to detect DNA when present in the sample), which can confound management decisions (Darling et al. 2021). These errors commonly derive from suboptimal assay parameters and poor assay design that result in ambiguous results (high cycle threshold [Ct] values in the case of qPCR and aberrant positive wells/pixels in the case of dPCR). The primary objective of this study was to develop a multiplex dPCR assay that can detect both the Asian swamp eel and bullseye snakehead from eDNA samples and provide data on eDNA samples (both controlled and natural) to demonstrate the utility for using the technology for monitoring efforts in Florida. Because of the similar habitat preference for both *C. aurolineata/marulius* and *M. albus/javanensis*, the development of a multiplex dPCR assay for these two species is practical in that analyzing eDNA samples from this habitat type will allow monitoring of two species simultaneously regardless of what the target species of interest is and will allow for a cost‐effective (relative to physical surveys/traditional monitoring protocols that require multiple people and more time to obtain data) means to determine where overlap of these two species occurs.

## 2 Materials and Methods

### 2.1 Animal Tissue Source and DNA Extraction

Animal tissue was harvested from BS and ASE specimens donated by the Florida Fish and Wildlife Conservation Commission acquired through invasive species removal efforts. Collection of live specimens for experiments was covered by UF IACUC protocol number 202200000027; FWC EXOT‐23‐63a; FWC EXOT‐23‐83. Euthanasia of the organisms used for this part of the study is covered by the above‐mentioned IACUC and performed according to the AVMA (2020) guidelines. Experimentation on live animals was also covered under the above‐mentioned IACUC.

Muscle tissue (100 mg) was excised from each specimen and transferred to a 1.5 mL microcentrifuge tube. Total DNA was extracted using the DNeasy Blood & Tissue Kit (Qiagen) according to the manufacturer's instructions but without macerating the tissue and using a lysis time of 24 h at 56°C. DNA concentration and purity were measured for each extraction using a NanoDropLite spectrophotometer (ThermoFisher). Extractions were then diluted to 25 ng/μL for running PCR assays.

### 2.2 Assay Design

For assay design, the cytochrome *c* oxidase subunit I (COI) barcoding region (5′‐half) was selected due to high levels of variability among species, allowing for development of species‐specific assays, which avoid cross‐amplification. Template obtained from BS and ASE was screened using the universal COI primers LCO1490 (forward) and HCO2198 (reverse) from Folmer et al. (1994). Reactions were run in volumes of 25 μL and were comprised of 5× GoTaq Flexi Buffer (Promega, Madison, Wisconsin, USA), 25 mM MgCl_2_, 200 μM dNTPs, 0.5 μM of forward and reverse primer, 2% PVP‐40, 1 U GoTaq Flexi DNA polymerase (Promega, Madison, Wisconsin, USA), and 2 μL of DNA template with sterile water to increase the final reaction volume to 25 μL. The utility of these primers for the fish species of interest was previously unknown, so insect DNA extract from a planthopper, *Pelitropis rotulata* Van Duzee, 1908 (Insecta: Hemiptera: Auchenorryhcna: Tropiduchidae), was used as a positive control. Thermal cycling conditions were as follows: initial denaturation at 95°C for 2 min followed by 35 cycles of denaturation at 95°C for 30 s, annealing for 30 s at 40°C, and extension at 72°C for 1 min 30 s, followed by a final extension at 72°C for 5 min (Table 1). Products obtained from PCR reactions were run on 1.5% agarose gel stained with GelRed (Biotium) and amplicons of the correct size, relative to the positive control, were sent for Sanger sequencing (Eurofins Genomics, Louisville, Kentucky, USA).

**TABLE 1:** Primers and probes used for generating COI sequences and running qPCR and dPCR assays for optimization and developing the multiplex assay with corresponding critical PCR parameters.

Species	Oligo name	Strand	Sequence (5′ ➔3′)	Annealing	Extension	Product size
Universal	LCO1490	Sense	GGTCAACAAATCATAAAGATATTGG	40°C	1 min 30 s	750 bp
	HCO2198	Antisense	TAAACTTCAGGGTGACCAAAAAATCA	40°C	1 min 30 s	
Bullseye snakehead (BS)	BS_COIF	Sense	GTACCAAACACCCCTATTTG	58°C	1 min	80 bp
	BS_COIR	Antisense	GCTAGTACTGGGAGTGAG	58°C	1 min	
	BS_probe	Sense	TCACCGCCGTACTTCTTCTCC	58°C	1 min	
Asian swamp eel (ASE)	ASE_COIF	Sense	GGGACGACCAAATCTATA	58°C	1 min	81 bp
	ASE_COIR	Antisense	CCGATTATAATAGGCATTACTA	58°C	1 min	
	ASE_probe	Sense	AACCGCTCACGCCTTCATTAT	58°C	1 min	

Sequence data were assembled using DNA Baser (Version 4.36) (Heracle BioSoft SRL, Pitesti, Romania) and aligned using Clustal*W* as part of the MEGA7 package (Kumar et al. 2016). Sections of 100 bps displaying variability among the two fish species were selected and uploaded to OligoArchitect Online (Sigma‐Aldrich) (https://www.oligoarchitect.com/LoginServlet) using the “Dual‐Labeled Probe” tab. In addition, publicly available sequences of closely related taxa and other species potentially present in Florida aquatic habitats that could represent contamination were also included to verify an adequate number of SNPs were present as oligonucleotide binding sites that would eliminate the risk of cross‐amplification (Figure S1). Each resulting assay was subsequently purchased as a TaqMan MGB (minor groove binder) probe with a 5′ FAM label and 3′ nonfluorescent quencher (NFQ) for optimization along with corresponding primers. Each assay was screened against its corresponding fish species with species‐specific primers only by standard PCR using a gradient to determine optimal annealing temperatures for the assay. For the gradient PCR, reactions were performed using the same concentrations as the initial PCR assays listed above with a gradient from 49°C to 60°C. Each resultant assay was labeled according to abbreviated common names of the fish species for ease of labeling and presentation; bullseye snakehead species complex assay = BS and Asian swamp eel species complex assay = ASE.

### 2.3 qPCR and dPCR Optimization

All assays designed were screened against the original template used to generate sequences that resulted in the corresponding assays. Each assay was screened in triplicate against the template for the corresponding fish species (representing positive controls) and screened in triplicate against the other fish species template (negative controls) to ensure no cross‐amplification occurs. All assays were run on a QuantStudio 6 Flex qPCR system (Applied Biosystems, ThermoFisher Scientific). Reactions were performed in volumes of 20 μL and comprised of 10 μL of TaqMan Universal Master Mix II with UNG, 10 μM for each oligonucleotide (forward primer, reverse primer, and probe), 10% polyvinylpyrrolidone (PVP‐40), 1 μL of DNA template with sterile dH_2_O added to reach a final volume (20 μL). Thermal cycling conditions for qPCR assays were as follows: initial hold at 50°C for 2 min, initial denaturation at 95°C for 10 min followed by 35 cycles of denaturation at 95°C for 15 s and annealing/extension at 56°C for 1 min.

Amplicons from the gradient PCR for both fish species were cloned using the pGEM‐T Easy Vector kit (Promega) following the manufacturer's instructions. The cloned vectors were then transformed into NEB Turbo Competent *E. coli* (New England BioLabs) and plated on Lysogeny broth (LB) plates containing 100 mg/mL of ampicillin (Alkali Scientific, Ft. Lauderdale, FL), 10 mg/mL X‐Gal, and 8 mg/ML IPTG in solution (AG Scientific, San Diego, CA). Plates were incubated overnight and transformed colonies were screened for the clones with correct inserts using M13F/M13R primers. Clones with an insert of the correct size were incubated at 37°C overnight on a shaker at 250 rpm in 20 mL of LB broth containing 100 mg/mL of ampicillin (Alkali Scientific, Ft. Lauderdale, FL). Finally, plasmids were extracted using a QIAprep Spin Miniprep Kit (Qiagen) per the manufacturer's instructions and sent for Sanger sequencing (Eurofins Genomics) to confirm identity of the inserts. Plasmid eluate was subsequently diluted to 10^7^ copies/μL followed by a serial dilution to 10^1^ copies/μL.

Serially diluted plasmids for BS and ASE were subsequently run with the corresponding assay on the QuantStudio Absolute Q Digital PCR System (dPCR; ThermoFisher Scientific) to establish optimal dilution concentrations. In addition, the eluate from the tissue extraction for BS and ASE was diluted to 25 ng/μL, then subsequently serially diluted (10:1) three times and screened with the corresponding assays to determine the optimal concentration for total DNA samples.

### 2.4 Multiplex Optimization

The multiplex assay was purchased directly from ThermoFisher. The 5′‐ends of each species‐specific probe were labeled as VIC‐BS and FAM‐ASE. Both assays were tagged on the 3′ end with MGB‐NFQ quencher. Plasmid standards and samples for each fish species with the optimal concentrations were screened using the multiplex assay. In addition, samples with optimal concentrations for both fish species were mixed to reflect the possibility of an eDNA sample containing multiple targets.

All reactions were run in volumes of 9 μL and comprised of 1.8 μL Absolute Q Master Mix (5×), 0.45 μL dPCR assay (20×), 1 μL DNA template and the remaining volume made up with UltraPure water. Thermal cycling conditions were as follows: 10 min initial denaturation at 95°C followed by 25 cycles of denaturation for 15 s at 95°C and annealing/extension for 1 min at 58°C. All reactions were run on the QuantStudio Absolute Q Digital PCR System (ThermoFisher Scientific). Limits of detection were established by diluting the DNA extract of each species/complex to 25 ng/μL then serially diluting to 0.000025 ng/μL and screening each dilution using the above‐mentioned protocol/parameters.

### 2.5 In Vitro Assessment of Assays Against Other Invasive Species

In vitro screening of other invasive species of interest in Florida to ensure no cross‐amplification occurs with the newly designed assays included: Burmese python ( *Python bivittatus* ), North African python (*Python seabae*), *Boa constrictor* , rainbow boa ( *Epicrates cenchria* ); Argentine black and white Argetine tegu (*Salvator merinae*), red tegu (*Tupinanbis rufecens*), gold tegu (*Tupinambis teguixin*), Nile monitor ( *Varanus niloticus* ), Asian water monitor ( *Varanus salvator* ), and spectacled caiman ( *Caiman crocodilus* ). Specimen vouchers of these species currently available had total DNA extracted using the protocol outlined above and were diluted to 25 ng/μL and screened with the assays developed for the BS and ASE using the qPCR protocol outlined above. In addition to these, a common invasive and present in the field sites in this study, the Mayan cichlid (*Mayaheros uropthalmus*), was included. Finally, vouchers of the American crocodile (*Crocodylus actutus*) and American alligator ( *Alligator mississipiensis* ) were also included and screened.

### 2.6 Validation of Assay on eDNA Controls

Controlled eDNA water samples were generated by placing a live bullseye snakehead (72.8 cm total length, 2.8 kg) in an opaque, black storage tote filled with tap water. Before filling with water and placing the fish within, the container was cleaned with a 10% bleach solution, rinsed with 70% ethanol and a final wash of distilled water to ensure sterile surface conditions. Autoclaved, glass Nalgene bottles were used to collect water samples of 1 L in volume. A baseline, negative control was collected before fish placement in water. After placing the fish in the water, 1 L samples were collected at time intervals of 5 min, 30 min, 1 h, 2 h, and 3 h. Water samples were stored in a cooler on ice and transported to the lab to be processed using the protocol outlined above. Any samples with a concentration above 1 ng/μL were diluted to 1 ng/μL with sterile dH_2_O to avoid potential oversaturation of the dPCR chips (100% positive wells).

### 2.7 Validation of Assay on eDNA Samples

For validating the BS‐assay, a canal was selected in Coral Springs, FL (26.245295 N, −80.270105 W) where at least one specimen of BS has been observed on multiple occasions (Figure S2). Glass bottles were filled with 1 L of water from the canal at three separate locations. Glass bottles were completely submerged (the tip of the bottle was at least 5 cm from the water surface) when collecting water samples to avoid neuston/particles that can contaminate samples. This sampling was replicated at the same canal (total of two replications each with three samples per replicate). Water was transported on ice to the lab and processed. Each water sample was filtered through a 0.22 μM nitrocellulose membrane (Merck Millipore Ltd., Billerica, MA) using a sterile filter holder (Nalgene, Rochester, NY) and a vacuum pump (model no. 1HAB‐25‐M100X, Gast). The full liter of each sample was processed, and the filters were dried for five minutes. Filters were stored at −20°C until DNA extraction. DNA was extracted from each filter using the SPINeasy DNA Kit for Water (MP Biomedicals LLC, USA) according to the manufacturer's instructions. Extracted DNA was quantified using a NanoDrop Lite spectrophotometer (ThermoFisher Scientific). Extractions were then tested using the optimized multiplex assay following the protocol described earlier.

For validating the ASE‐assay, a canal was selected in Pembroke Pines, FL (26.005416 N, −80.390283 W) where a specimen of *M. albus* had been observed (Figure S3). Water samples were collected approximately one week after the specimen had been observed/collected in the parking lot. Glass bottles were filled as previously described with 1 L of water from the canal collected at three distinct locations including the southwest (SW) end, the northeast (NE) end, and the southeast (SE) corner. Water was transported on ice to the lab and processed the same as water samples in the previous sections. Final eluate for all samples was screened using the optimized multiplex assay listed previously.

Three negative eDNA controls were included and comprised three separate ponds located at the University of Florida's Fort Lauderdale Research and Education Center (FLREC) that are remote from and not connected to canals in the surrounding urban environment. In addition, a distilled water control was also included in the analysis. Negative eDNA controls were collected and processed the same as the field samples listed above.

## 3 Results

### 3.1 Assay Design and qPCR Optimization

The initial PCR reactions for both fish species using the universal primer set resulted in positive amplification. For each species, a raw 691 bp sequence was obtained and when entered in BLAST, matched the corresponding species ( *C. marulius* and *M. albus* ) in GenBank. Sequences for each species were aligned, trimmed, and deposited in GenBank; *C. marulius* = (PQ736517) and *M. albus* = (PQ736518), for final product length of 673 and 678 bp, respectively. Sequence data obtained for *C. marulius* used in this study showed a 100% nucleotide match for *C. marulius* sequences present in GenBank (Figure S4) and sequence data for *M. albus* used in this study demonstrated the highest nucleotide identity to *M. albus* based on available data; however, it was not a 100% match (1.8% variance) (Figure S4). Resulting primers and probes for each respective fish species are presented in Table 1 along with optimal annealing temperatures and times. Both assays were found to run optimally at 58°C for 1 min based on the gradient PCR. When run by qPCR with the TaqMan probe included, both assays were successful in amplifying all three replicates of the target species (average Ct of 19.10 ± 1.5 and 19.10 ± 0.12 for BS and ASE, respectively) and without cross‐amplification (No Ct) of non‐target species (Table 2).

**TABLE 2:** dPCR results for the multiplex assay to validate function and verify the absence of cross‐amplification for fish species analyzed in this study based on optimal sample concentrations for plasmids and DNA extract; CI = confidence intervals.

	Plasmid (10^3^ copies/μL)		Sample (0.25 ng/μL)	
	VIC‐BS	FAM‐ASE	VIC‐BS	FAM‐ASE
Bullseye snakehead (BS)				
Positives wells (%)	29.68	0.0	28.7	0.0
Total wells	20,364	20,364	20,411	20,411
Conc. cp/μL	815.23	0.0	783.05	0.0
95% CI	20.4	n/a	19.89	n/a
Asian swamp eel (ASE)				
Positives wells (%)	0.0	36.02	0.0	21.69
Total wells	20,380	20,380	20,455	20,455
Conc. cp/μL	0.0	1033.82	0.0	565.86
95% CI	n/a	23.57	n/a	16.45

### 3.2 Optimization for Concentration Levels, Threshold Establishment, and dPCR Assay Validation

Serially diluted plasmids for BS successfully amplified at all concentrations using the FAM‐labeled probe. Digital PCR chips were fully or nearly fully saturated (approximately 95–100% positive wells) at concentrations of 10^7^ copies/μL to 10^4^ copies/μL. At the 10^3^ copies/μL concentration, approximately 30.2% of the wells displayed successful amplification, with 3.3% and 0.5% successful amplification observed for 10^2^ and 10^1^ copies/μL concentrations, respectively. The optimal concentration for the BS plasmids was 10^3^ copies/μL, having a 95% confidence interval (CI) value of 20.66 (Table S1).

Serially diluted plasmids for ASE successfully amplified at all concentrations using the FAM‐labeled probe. Digital PCR chips were fully or nearly fully saturated (approximately 98–100% positive wells) at concentrations of 10^7^ copies/μL to 10^4^ copies/μL. At the 10^3^ copies/μL concentration, approximately 27.0% of the wells displayed successful amplification, with 5.7% and 1.1% successful amplification observed for 10^2^ and 10^1^ copies/μL concentrations, respectively. The optimal concentration for the ASE plasmids was 10^3^ copies/μL, having a 95% confidence interval (CI) value of 19.34 (Table S1).

**FIGURE 2:**
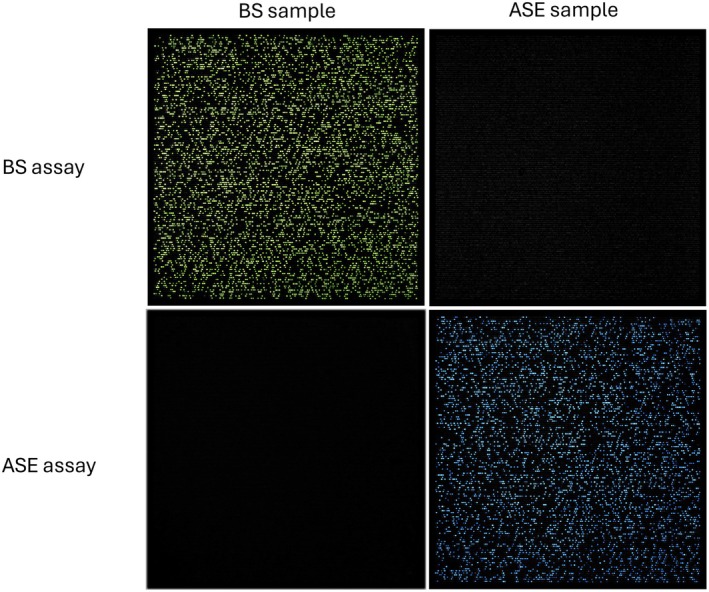
Images of dPCR chips showing amplification of bullseye snakehead (BS)and Asian swamp eel (ASE) with the corresponding assays; green = VIC reporter, blue = FAM reporter, and black = no amplification.

Final eluate from the total DNA extractions of BS also had successful amplification for all concentrations. At concentrations of total DNA of 25 ng/μL and 2.5 ng/μL, chips were nearly fully saturated (95%–98% of wells reading as positive) while concentrations of 0.25 ng/μL and 0.025 ng/μL total DNA yielded approximately 28% and 4% positive wells. The calculated copy number for concentrations of 0.25 ng/μL and 0.025 ng/μL was 753.14 (19.43 95% CI) and 82.21 (5.82 95% CI), respectively (Table 3). Final eluate from the total DNA extractions of ASE also had successful amplification for all concentrations. At concentrations of total DNA of 25 ng/μL and 2.5 ng/μL, chips were nearly fully saturated (95%–98% of wells reading as positive) while concentrations of 0.25 ng/μL and 0.025 ng/μL total DNA yielded approximately 26% and 2% positive wells. The calculated copy number for concentrations of 0.25 ng/μL and 0.025 ng/μL was 683.09 (18.34 95% CI) and 56.95 (4.8 95% CI), respectively (Table 3). Based on optimal concentrations established for both the plasmids and DNA extract for both BS and ASE, the fluorescence threshold for scoring positives with the multiplex assay was 1000 for ASE and 500 for BS. Finally, the mixed sample containing 50% template from BS and 50% template from ASE yielded positive results for both BS and ASE relative to their corresponding assay with no cross‐amplification detected (Figure 2).

**TABLE 3:** Limit of detection using dPCR for the species analyzed in this study.

Species	Concentration (ng/μL) of total DNA						
Bullseye snakehead	25	2.5	0.25	0.025	0.0025	0.00025	0.000250
Positive wells (%)	99.9	96.0	19.3	2.0	0.2	0.02	0.004
Total wells	20,478	20,478	20,469	20,460	20,463	20,471	20,448
Conc. cp/μL	15824.2	7431.7	495.0	46.6	4.5	0.3	0.1
95% CI	938,997	153,156	15,16	4,5	1,2	0,1	0,1
Precision %	6.3	2.1	3.2	10.2	36.3	210.1	609.9
Asian swamp eel							
Positive wells (%)	99.9	94.4	22.9	1.7	0.2	0.05	0.03
Total wells	20,477	20,474	20,478	20,452	20,458	20,448	20,460
Conc. cp/μL	16710.7	6679.4	601.9	39.8	4.0	1.3	0.8
95% CI	1131,1213	129,32	17,18	4,4	1,2	1,1	0,1
Precision %	7.3	2.0	2.9	11.1	39.3	80.6	109.8

The limit of detection for each species is between 0.000025 ng/μL total DNA and 0.0000025 ng/μL total DNA. Both species yielded positive reactions even at the lowest dilutions (Table 3).

### 3.3 In Vitro Assay Validation

All invasive and native species isolates selected and screened against the ASE and BS assays developed in this study tested negative by qPCR (Table S2). Reactions were run in triplicate and all three reactions for each species were negative.

### 3.4 Validation of Assay on eDNA Controls

Under controlled conditions, positive results were obtained at all time points after immersion of the fish in the water (Table 4). The highest level detected was at 1 h with an estimate of 1189.01 copies/μL, then a slight drop at the 2 h time point and a significant drop at the 3 h time point (Table 4). The baseline control of the water before introduction of the organism tested negative (Table 4). At all time points, except the baseline control, only 500 mL of the 1 L water samples were able to be analyzed due to cellular debris clogging the filter.

**TABLE 4:** dPCR results for controlled eDNA analysis of Bullseye snakehead.

Time point	% (+) wells	Total wells	Conc. (cp/μL)	95% CI
Baseline	0.0	20,461	0.0	0.097, 0.690
5 min	26.2	20,465	703.6	18.650, 19.158
30 min	32.1	20,461	897.7	21.567, 22.098
1 h	40.2	20,441	1189.0	25.720, 26.288
2 h	37.4	20,456	1084.0	24.240, 24.795
3 h	16.2	20,468	408.6	13.699, 14.174

### 3.5 Validation of Assay on eDNA Samples

**FIGURE 3:**
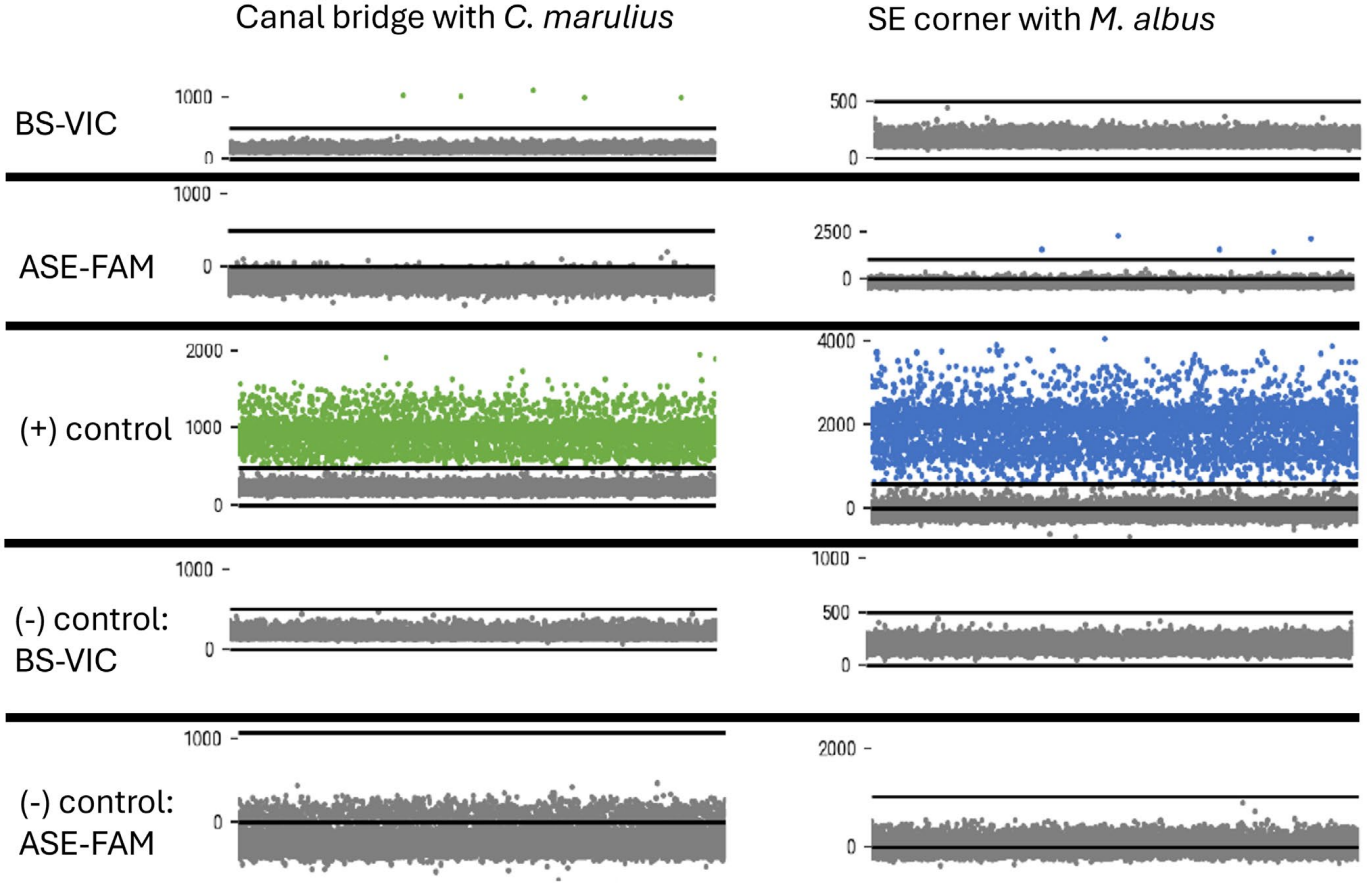
Fluorescence data obtained from eDNA samples collected at locations with observed specimens of bullseye snakehead (BS) (left) and Asian swamp eel (ASE) (right).

Samples taken from the canal with an observed specimen of BS on the first replicate (August 20, 2024) tested positive near the road (1.81 copies/μL) and at the mouth of the canal (0.23 copies/μL) while the sample taken under the bridge tested negative (Table 5). All samples taken at this location tested negative for ASE (Table 5). The second sampling (September 3, 2024) yielded positive results at all points (near road, under bridge, and at the mouth of the canal) for BS with the highest concentration near the road (1.58 copies/μL), a lower concentration detected under the bridge (0.57 copies/μL), and the lowest concentration detected at the mouth of the canal (0.23 copies/μL) (Table 5). All samples from this replicate tested negative for ASE (Table 5). For both replicates, all negative water controls tested negative and all positive controls amplified with levels of fluorescence consistent with fluorescence detected in samples positive for BS (Table 5). Positive reactions for BS in eDNA samples were at a level consistent with positive control fluorescence (Figure 3).

**TABLE 5:** Digital PCR results for eDNA samples taken at locations with documented sightings of Bullseye snakehead (BS) and Asian swamp eel (ASE).

	VIC‐BS				FAM‐ASE			
	Total	Pos.	Conc.	95% CI	Total	Pos.	Conc.	95% CI
Canal—BS rep. 1								
Road	20,450	16	1.81	0.7, 1.2	20,450	0	0.0	n/a
Bridge	20,455	0	0.0	n/a	20,455	0	0.0	n/a
Mouth	20,456	2	0.23	0.2, 0.7	20,456	0	0.0	n/a
Canal—BS rep. 2								
Road	20,461	14	1.58	0.4, 0.9	20,461	0	0.0	n/a
Bridge	20,461	5	0.57	0.2, 0.7	20,461	0	0.0	n/a
Mouth	20,414	2	0.23	0.2, 0.7	20,414	0	0.0	n/a
Canal—ASE								
NE end	20,473	0	0.0	n/a	20,473	5	0.57	0.3, 0.8
Corner	20,468	0	0.0	n/a	20,468	5	0.57	0.3, 0.8
SW end	20,467	0	0.0	n/a	20,467	1	0.11	0.1, 0.7
(+) control	20,411	5858	783.05	19.89				
Pond 1 (−) control	20,466	0	0.0	n/a	20,466	0	0.0	n/a
Pond 2 (−) control	20,463	0	0.0	n/a	20,463	0	0.0	n/a
Pond 3 (−) control	20,441	0	0.0	n/a	20,441	0	0.0	n/a
DI water (−) control	20,474	0	0.0	n/a	20,474	0	0.0	n/a

Samples taken from the canal where the specimen of the ASE was observed resulted in positive reactions at the SW end (0.11 copies/μL) and SE corner (0.57 copies/μL) and a negative reaction at the NE end (Table 5). All samples from this location tested negative for BS (Table 5). Positive reactions for ASE in eDNA samples were at a level consistent with positive control fluorescence (Figure 3). All negative water controls (distilled water and pond water at FLREC) tested negative (Table 5) and positive controls amplified with levels of fluorescence consistent with fluorescence detected in samples positive for ASE (Table 5).

## 4 Discussion

The development of a multiplex assay for these two important invasive fish species provides a valuable tool for natural resource managers to simultaneously monitor for the presence (or likely absence) of both BS and ASE in urban canals and natural ecosystems in Florida. Despite the fact that these two species largely do not overlap in distribution, there is a slight overlap of the ranges in northern Miami‐Dade County, southern Broward County, and northern Palm Beach County where both species can be found (Figure 1). As reporting of introduced species often occurs post‐establishment, particularly for nonnative aquatic species due in part to variable survey efforts resulting from often resource‐intensive sampling methodologies (Bailey et al. 2020) and given the interconnectedness of canal systems and natural aquatic ecosystems in southern Florida, it is likely that the current distributions of BS and ASE are underestimated or, if accurate, will change over time as both species spread, resulting in greater regions of overlap. A multiplex assay capable of simultaneous testing for the presence of BS and ASE from a water sample can provide managers with an effective monitoring tool, requiring fewer resources compared with traditional methods. Further justified by the fact that both ASE and BS inhabit lakes, canals, and swamps with little or no flow, making it an ideal habitat for eDNA monitoring to supplement conservation and/or regulatory efforts.

The assay reported herein and demonstration of its utility on eDNA samples for both species without cross‐amplification provides a critical tool for monitoring programs and a cost‐effective means to evaluate removal efforts. Additionally, all sites sampled in this study (including the pond controls) contained large populations of nonnative Mayan cichlid and butterfly peacock bass ( *Cichla ocellaris* ), yet no ambiguous results were obtained that would indicate cross‐amplification with common and widespread fish species in Florida. Furthermore, a specimen of *M. uropthalmus* was collected, DNA extracted, and screened with the assay, resulting in no detection (Table S2).

The concerns by Jerde (2021) regarding false positives in eDNA samples are valid as ambiguous results can have significant negative consequences for invasive species management, including application of resources where they are not needed; therefore, detracting resources from efforts in areas where species actually are present, which may delay efforts and exacerbate issues. Two critical factors were addressed in this study to ensure the reliability of results. The first being in assay design, where a highly variable gene (COI) was selected and analyzed with close relatives of the target organisms as well as taxa likely to be present in locations sampled for the target organisms to ensure an adequate number of SNPs were present at binding sites that will eliminate or reduce cross‐amplification. The second factor considered was the dPCR parameters which were modified from factory default settings to further reduce the potential for ambiguous results (lowering the cycle number to 25 cycles rather than 40). Limits of detection established by Miller et al. (2024) for the equipment and protocol used in that study were used in this study to evaluate results in a controlled experiment. The limits of detection presented herein are consistent with those observed by Miller et al. (2024), where 0.25 ng appears to be the optimal concentration for establishing thresholds, and positives can still be obtained at very low concentrations, well beyond the capacity of qPCR to detect positives (Bahder et al. 2018). Generally, limits of detection are not of practical use for dPCR with regard to results interpretation. With dPCR, a single positive can be scored positive reliably provided it matches fluorescence thresholds (RFUs). Limits of detection are more practical for interpreting qPCR results, where it can be difficult to distinguish between true positives that are in low concentrations (high Ct values) versus non‐target fluorescence (primer dimer, probe degradation, inhibitors, etc.). These limits are certainly impractical when interpreting field samples using dPCR because often, trace amounts are expected, so many samples are needed to obtain results, and detection limits by equipment can be offset by proper sampling protocols.

Similarly, *M. albus* binding sites were highly variable when compared with other species except for *M. javanensis* , which differs by about 7.3% from *M. albus* but only has one SNP in the reverse primer region. However, when compared with *C. marulius* and other species analyzed that are present in Florida, the assay for *M. albus* possesses many SNPs that will eliminate cross‐amplification.

The similarity of *C. marulius* to *C. aurolineata* and *M. albus* to *M. javanensis* at the molecular level means that these assays will not be able to distinguish between these taxa. While taxonomic error or ambiguity may sow confusion as to the identity of what is present in an area, these differences may be irrelevant for invasive management because whether they are distinct populations or distinct species. While different clades/species/haplotypes may result in different environmental tolerances, the behaviors among these closely related taxa are similar, meaning sampling (with regard to eDNA) would be conducted the same regardless of species present and detection would result in sampling for the physical specimens and ultimate removal, regardless of the taxon collected. It is important to note that the ASE has multiple isolated populations in Florida. It has been documented that there is genetic variation within species of *Monopterus* in Florida (Collins et al. 2002). The assays designed in this study will amplify closely related species (within the species complex), so the likelihood of them failing to detect a specific haplotype is extremely low since the level of genetic variance among populations will be lower than what is observed among species. While this is a possible issue, from a practical perspective, it is not a concern for implementing this technology. Future efforts will seek to collect/obtain specimens of each haplotype to ensure there are issues with detection of all variants. Because of the stringent nature of the assay/experimental parameters, if a SNP/SNPs occur at assay sites among haplotypes, the protocol can be modified (higher cycle number, lower annealing, etc.) to be more flexible and allow for amplification of targets that are not a 100% match at the assay binding sites.

While the high level of SNPs observed at binding sites is a crucial component to specific assays that do not yield ambiguous results, the reagents used are resilient enough to tolerate some number of SNPs so that assays that mismatch with a target species can still amplify. In this case, the reduction of cycle number can eliminate these ambiguous results. Because of mismatches with assays and template, amplification may be significantly less efficient, resulting in positives that will be lower in fluorescence than true positives. A well‐designed assay can detect target DNA in qPCR and dPCR reactions easily at 25 cycles, so lowering the conditions to only have 25 cycles can cut off the reaction before mismatched assays can amplify to detectable levels of non‐target DNA while still amplifying target DNA to detectable levels, even when a single molecule is present in a given well. Because of the ability of dPCR to amplify/detect single copies of target DNA, the sensitivity of the assay is not influenced by cycle number (i.e., if you have 1 copy of target DNA in a sample and run it on dPCR, your copy estimate will always be 1, whether it is run at 25 cycles or 50 because only a single well is partitioned). With lower cycle numbers, you can reliably detect trace amounts of DNA while eliminating false positives that result from cross‐amplification with non‐target organisms, probe degradation, or secondary structures (Table S3). With qPCR, detecting trace amounts of DNA, while possible, is inconsistent due to the large volume of the reaction sizes compared with dPCR, making it not suitable or significantly less reliable in eDNA analysis. While qPCR is generally more accessible and cheaper to run per sample (approximately $200 a sample for dPCR when factoring time and supplies versus $100 per sample for qPCR or $42 versus $12 based on raw supplies in this scenario, unpublished data), the difficulty in distinguishing results with high Ct values/inconsistencies with low copy amplification require replication (potentially significantly high replication) that will quickly make obtaining reliable qPCR results significantly more expensive than running dPCR assays (which technically, are approximately 20,000 separate PCR reactions/replications on a given sample). In practice, dPCR is well known to be more sensitive than qPCR. In one example, dPCR was able to detect target DNA of phytoplasmas at two dilution factors more than qPCR (Bahder et al. 2018). Other studies have demonstrated successful amplification of target DNA by dPCR where qPCR assays failed to give positive results (Mou et al. 2022, Herrera‐Blitman et al. 2025).

The findings in this study highlight the utility of a novel multiplex assay for two important invasive fish species in Florida and demonstrate its success in detecting target organisms in eDNA samples. Future efforts in this area will seek to significantly expand sampling and testing efforts across South Florida, integrate this detection protocol into monitoring programs, and subsequently utilize it in evaluating the efficacy of removal efforts.

## Author Contributions

**Melody Bloch:** data curation (equal), formal analysis (equal), investigation (equal), methodology (equal), validation (equal), writing – original draft (equal), writing – review and editing (equal). **Eric Suarez:** conceptualization (equal), project administration (equal), resources (equal), writing – original draft (equal), writing – review and editing (equal). **Melissa A. Miller:** funding acquisition (equal), project administration (equal), resources (equal), supervision (equal), writing – original draft (equal), writing – review and editing (equal). **Sergio A. Balaguera‐Reina:** conceptualization (equal), funding acquisition (equal), investigation (equal), methodology (equal), project administration (equal), resources (equal), supervision (equal), writing – original draft (equal), writing – review and editing (equal). **Cynthia A. Fussell Persaud:** data curation (equal), investigation (equal), validation (equal), visualization (equal). **Kevin A. Olejniczak:** data curation (equal), investigation (equal), validation (equal), visualization (equal). **Ericka E. Helmick:** data curation (equal), formal analysis (equal), investigation (equal), methodology (equal), validation (equal), visualization (equal), writing – original draft (equal). **Frank J. Mazzotti:** conceptualization (equal), funding acquisition (equal), project administration (equal), resources (equal), supervision (equal), writing – original draft (equal), writing – review and editing (equal). **Brian W. Bahder:** conceptualization (equal), formal analysis (equal), methodology (equal), project administration (equal), supervision (equal), writing – original draft (equal), writing – review and editing (equal).

## Funding

This work was supported by the South Florida Water Management District, 4600004856. UF/IFAS Invasion Science Research Institute, SEED grant.

## Disclosure

Benefit‐sharing statement: Benefits from this research accrue from the sharing of our data and results on public databases as described above.

## Conflicts of Interest

The authors declare no conflicts of interest.

## Supporting information


**Figure S1:** Sequence alignments for sections of COI gene highlighting assay binding locations (red) for *Channa marulius* (A) and *Monopterus albus* (B) relative to other closely related taxa and other species likely to be present where eDNA sampling of target organisms will occur.

**Figure S2:** Field site selected for eDNA sampling to validate protocol for detection of the bullseye snakehead ( *Channa marulius* ): (A) canal mouth, (B) canal bridge, and (C) canal road.

**Figure S3:** Field site selected for eDNA sampling to validate protocol for detection of the Asian swamp eel ( *Monopterus albus* ): (A) NE end, (B) SE corner, and (C) SW end.

**Figure S4:** Maximum likelihood phylogenetic trees showing relatedness of specimen of *Channa marulius* (A) and Asian swamp eel ( *Monopterus albus* ) (B) collected from Florida, USA, for use in assay design: red asterisk = sequence generated in this study.

**Table S1:** dPCR results for plasmid serial dilutions and sample dilutions for Bullseye snakehead (BS) and Asian swamp eel (ASE); % (+) and total = wells, Conc. = copies/μL, CI = confidence interval.
**Table S2:** In vitro assessment of assays developed in this study against common invasive and native species present in Florida ecosystems.
**Table S3:** dPCR results with selected targets run with different cycle numbers.

## Data Availability

Molecular data generated in this study are available on GenBank and have been released (National Center for Biotechnology Information (https://www.ncbi.nlm.nih.gov/nuccore/)). Accession numbers for associated data are PQ736517 and PQ736518. All other data associated with the study are presented within the manuscript.
