# Supplementary figures and images for: Development of a Duplex Digital PCR and Validation on eDNA Water Samples for Monitoring of the Asian Swamp Eel (Monopterus albus/Javanensis) and Bullseye Snakehead (Channa aurolineata/Marulius) in Florida, USA, Freshwater Ecosystems
Source: Ecol Evol. 2026 Feb 17;16(2):e73088. doi: 10.1002/ece3.73088 (PMC12912945; doi:10.1002/ece3.73088)

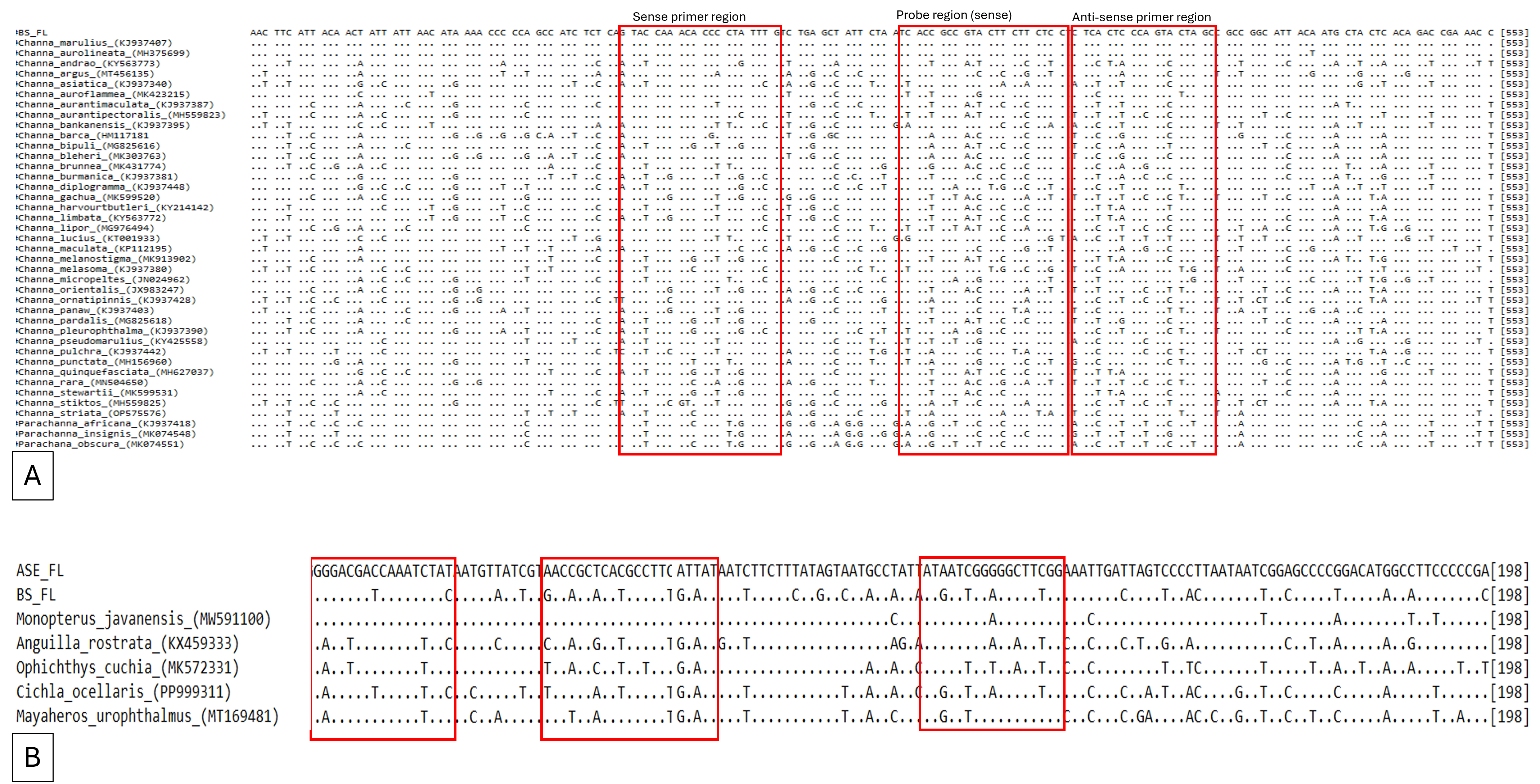

Supplement: Supplementary file 1 — Figure S1: Sequence alignments for sections of COI gene highlighting assay binding locations (red) for Channa marulius (A) and Monopterus albus (B) relative to other closely related taxa and other species likely to be present where eDNA sampling of target organisms will occur. [file ECE3-16-e73088-s002.tif]

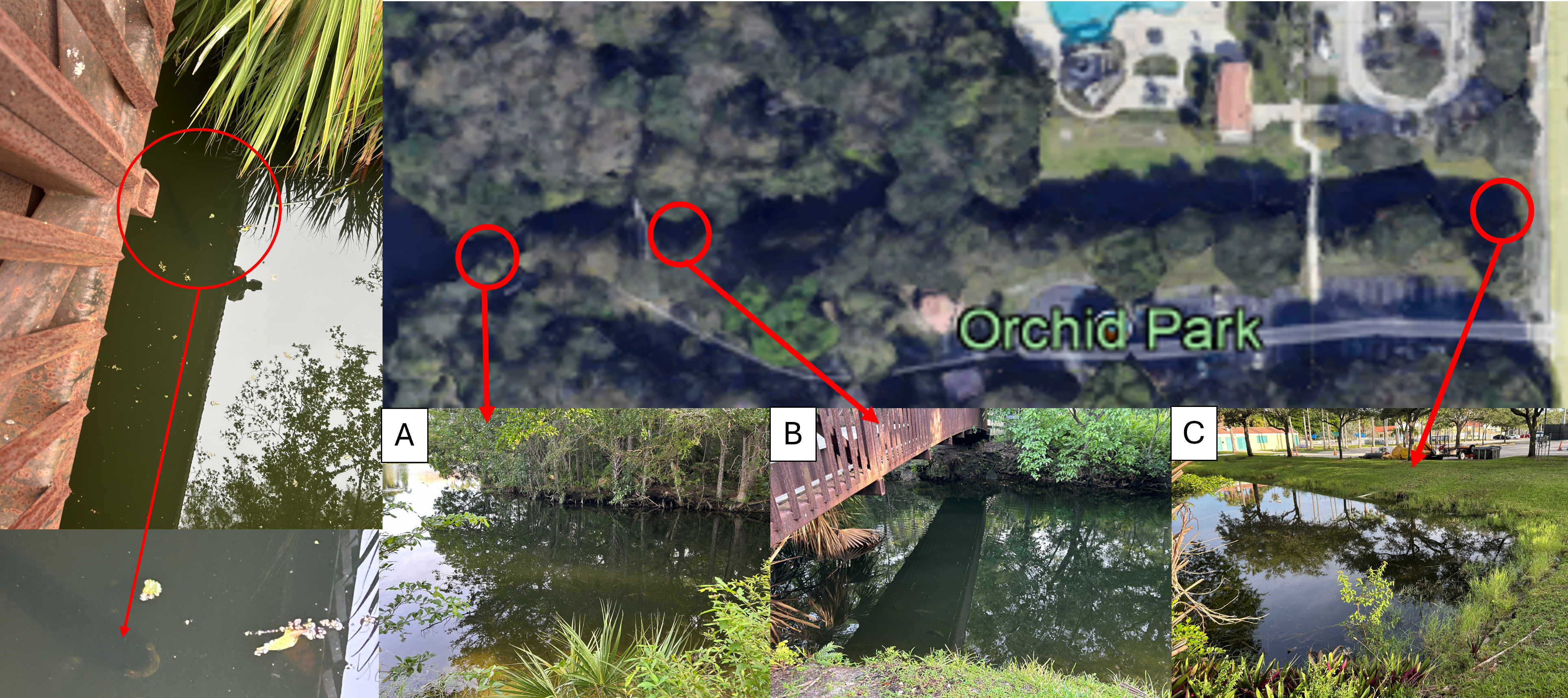

Supplement: Supplementary file 2 — Figure S2: Field site selected for eDNA sampling to validate protocol for detection of the bullseye snakehead ( Channa marulius ): (A) canal mouth, (B) canal bridge, and (C) canal road. [file ECE3-16-e73088-s001.tif]

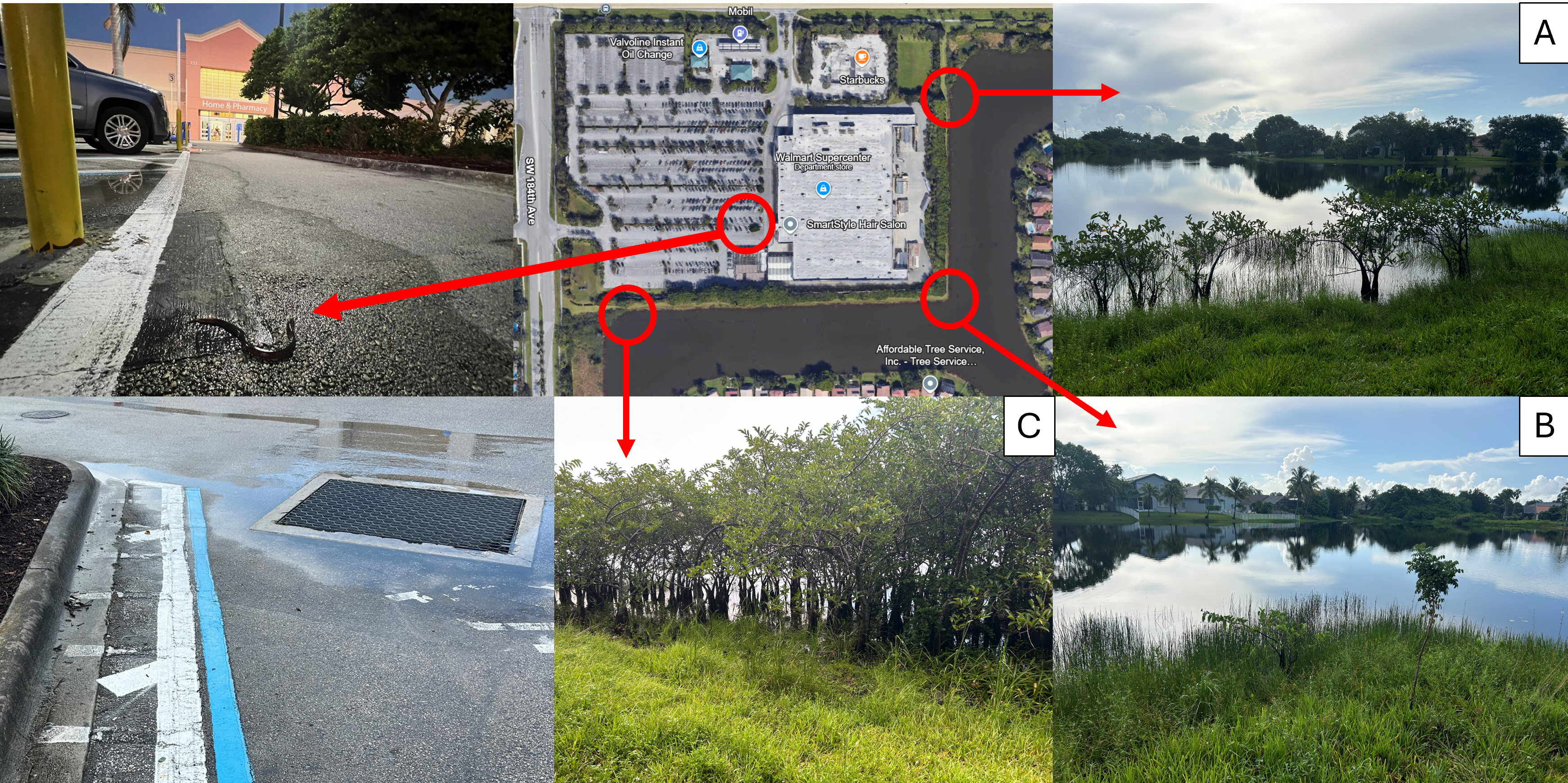

Supplement: Supplementary file 3 — Figure S3: Field site selected for eDNA sampling to validate protocol for detection of the Asian swamp eel ( Monopterus albus ): (A) NE end, (B) SE corner, and (C) SW end. [file ECE3-16-e73088-s003.tif]

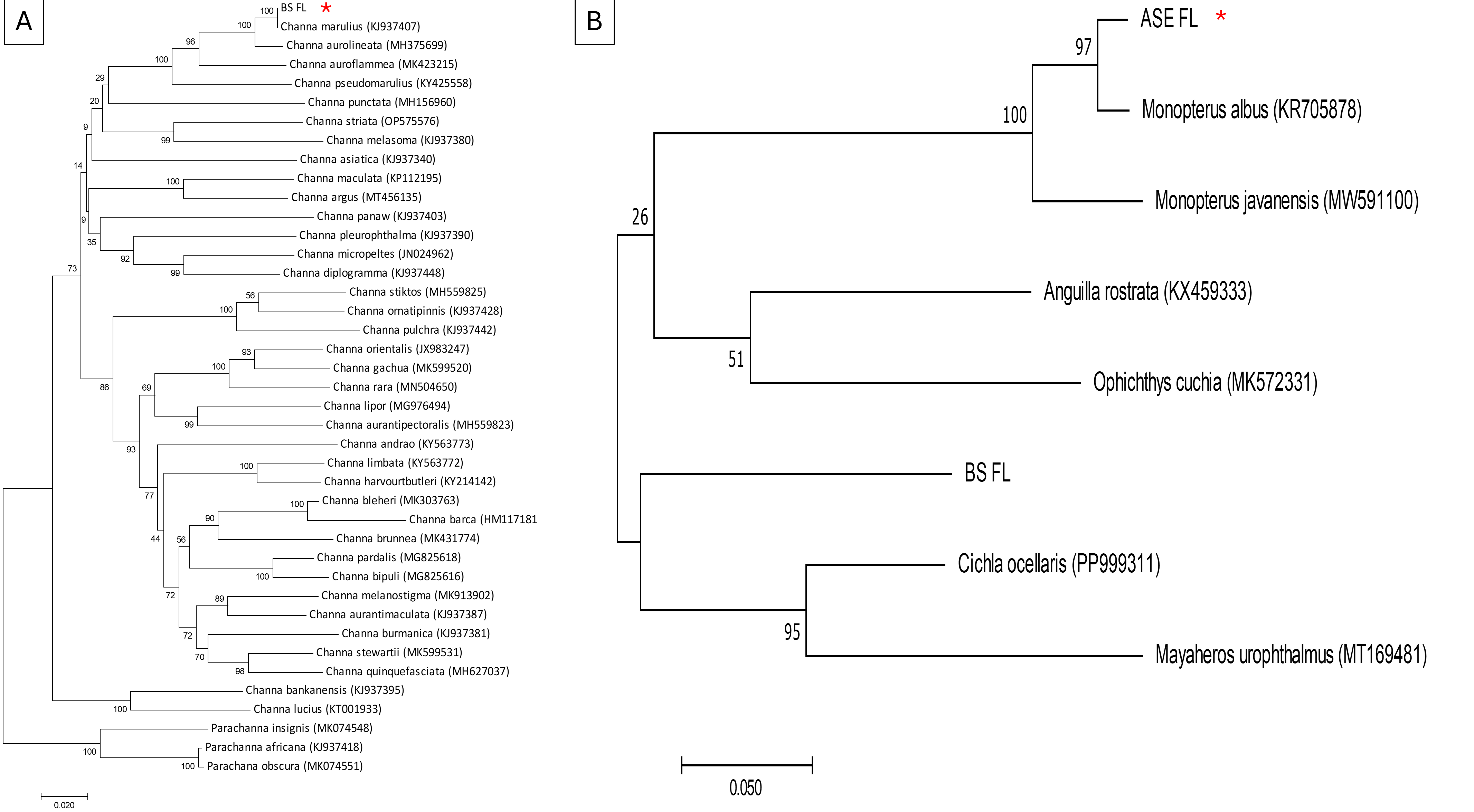

Supplement: Supplementary file 4 — Figure S4: Maximum likelihood phylogenetic trees showing relatedness of specimen of Channa marulius (A) and Asian swamp eel ( Monopterus albus ) (B) collected from Florida, USA, for use in assay design: red asterisk = sequence generated in this study. [file ECE3-16-e73088-s005.tif]
