# Supplementary material for: Development of a Duplex Digital PCR and Validation on eDNA Water Samples for Monitoring of the Asian Swamp Eel (Monopterus albus/Javanensis) and Bullseye Snakehead (Channa aurolineata/Marulius) in Florida, USA, Freshwater Ecosystems
Source: Ecol Evol. 2026 Feb 17;16(2):e73088. doi: 10.1002/ece3.73088 (PMC12912945; doi:10.1002/ece3.73088)
Supplement: Supplementary file 5 — Table S1: dPCR results for plasmid serial dilutions and sample dilutions for Bullseye snakehead (BS) and Asian swamp eel (ASE); % (+) and total = wells, Conc. = copies/μL, CI = confidence interval. Table S2: In vitro assessment of assays developed in this study against common invasive and native species present in Florida ecosystems. Table S3: dPCR results with selected targets run with different cycle numbers. [file ECE3-16-e73088-s004.docx]

**Supplementary Table 1.** dPCR results for plasmid serial dilutions and sample dilutions for Bullseye snakehead (BS) and Asian swamp eel (ASE); % (+) and total = wells, Conc. = copies/µL, CI = confidence interval.

| Concentration | VIC_BS | | | | FAM_ASE | | | |
| --- | --- | --- | --- | --- | --- | --- | --- | --- |
| Plasmid | % (+) | Total | Conc. | 95% CI | % (+) | Total | Conc. | 95% CI |
| 10^7^ copies/µl | 99.9 | 20436 | 16158.8 | 1007.6 | 100 | 20462 | 18830.0 | 1763.81 |
| 10^6^ copies/µl | 99.1 | 20418 | 11017.1 | 336.26 | 98.9 | 20360 | 10501.9 | 301.23 |
| 10^5^ copies/µl | 98.6 | 20428 | 9938.5 | 266.14 | 98.0 | 20449 | 9038.6 | 218.57 |
| 10^4^ copies/µl | 95.4 | 20336 | 7104.1 | 142.67 | 100 | 19439 | 22858.9 | 4115.05 |
| 10^3^ copies/µl | 30.2 | 20375 | 832.9 | 20.66 | 27.0 | 19825 | 728.7 | 19.34 |
| 10^2^ copies/µl | 3.3 | 20397 | 76.7 | 5.62 | 5.7 | 20059 | 136.8 | 7.68 |
| 10^1^ copies/µl | 0.5 | 20393 | 11.6 | 2.05 | 1.1 | 20170 | 24.9 | 3.11 |
| Sample |  |  |  |  |  |  |  |  |
| 25 ng/µl | 98.9 | 20330 | 10364.4 | 292.6 | 98.1 | 20452 | 9136.5 | 223.29 |
| 2.5 ng/µl | 94.1 | 20452 | 6564.3 | 125.9 | 94.8 | 20460 | 6822.0 | 113.44 |
| 0.25 ng/µl | 27.8 | 20437 | 753.1 | 19.4 | 25.6 | 20447 | 683.1 | 18.34 |
| 0.025 ng/µl | 3.5 | 20434 | 82.2 | 5.9 | 2.43 | 20410 | 57.0 | 4.8 |

**Supplementary Table 2**. *In Vitro* assessment of assays developed in this study against common invasive and native species present in Florida ecosystems*.*

|  | BS assay | | | ASE assay | | |
| --- | --- | --- | --- | --- | --- | --- |
| Species | Ct | Qty. (copies/µL) | Result | Ct | Qty. (copies/µL) | Result |
| Crocodilians |  |  |  |  |  |  |
| American alligator | No Ct | 0.0 | - | No Ct | 0.0 | - |
| American crocodile | No Ct | 0.0 | - | No Ct | 0.0 | - |
| Spectacled caiman | No Ct | 0.0 | - | No Ct | 0.0 | - |
| Snakes |  |  |  |  |  |  |
| Boa constrictor | No Ct | 0.0 | - | No Ct | 0.0 | - |
| Burmese python | No Ct | 0.0 | - | No Ct | 0.0 | - |
| North Afriacan python | No Ct | 0.0 | - | No Ct | 0.0 | - |
| Rainbow boa | No Ct | 0.0 | - | No Ct | 0.0 | - |
| Monitors |  |  |  |  |  |  |
| Asian water monitor | No Ct | 0.0 | - | No Ct | 0.0 | - |
| Nile monitor | No Ct | 0.0 | - | No Ct | 0.0 | - |
| Tegus |  |  |  |  |  |  |
| Black and White | No Ct | 0.0 | - | No Ct | 0.0 | - |
| Gold | No Ct | 0.0 | - | No Ct | 0.0 | - |
| Red | No Ct | 0.0 | - | No Ct | 0.0 | - |
| Fish |  |  |  |  |  |  |
| Mayan cichlid | No Ct | 0.0 | - | No Ct | 0.0 | - |

**Supplementary Table 3.** dPCR results with selected targets run with different cycle numbers.

|  | VIC-BS | | FAM-ASE | |
| --- | --- | --- | --- | --- |
|  | % positive wells | Conc. cp/µl | % positive wells | Conc. cp/µl |
| BS |  |  |  |  |
| 25 cycles | 2.66±0.1 | 62.5±1.8 | 0.0 | 0.0 |
| 30 cycles | 2.67±0.2 | 62.6±4.1 | 0.006±0.1 | 0.13±0.1 |
| 35 cycles | 2.63±0.2 | 61.7±3.9 | 0.03±0.1 | 0.79±0.1 |
| 40 cycles | 2.65±0.0 | 61.7±0.6 | 0.05±0.1 | 1.1±0.1 |
| ASE |  |  |  |  |
| 25 cycles | 0.0 | 0.0 | 2.01±0.1 | 47.0±1.6 |
| 30 cycles | 0.0 | 0.0 | 2.19±0.1 | 51.4±1.6 |
| 35 cycles | 0.0 | 0.0 | 2.17±0.1 | 50.8±3.4 |
| 40 cycles | 0.0 | 0.0 | 2.31±0.1 | 54.1±1.8 |
| Water control |  |  |  |  |
| 25 cycles | 0.0 | 0.0 | 0.0 | 0.0 |
| 30 cycles | 0.0 | 0.0 | 0.03±0.1 | 0.79±0.1 |
| 35 cycles | 0.0 | 0.0 | 0.03±0.1 | 0.79±0.1 |
| 40 cycles | 0.006±0.1 | 0.13±0.1 | 0.06±0.1 | 1.36±0.1 |
